# The impact of SARS-Cov-2 pandemic on mental health in medical staff

**DOI:** 10.1192/j.eurpsy.2023.1653

**Published:** 2023-07-19

**Authors:** C.-B. Bacaiteanu, B.-O. Bucatos, A. Romosan, M. Bondrescu, A.-M. C. Daescu, R. Romosan, I. Papava, R. Laza, L. Dehelean

**Affiliations:** 1 Neurosciences, Timisoara County Emergency Clinical Hospital; 2 Neurosciences; 3 Infectious Diseases, Victor Babes University of Medicine and Pharmacy Timisoara, Timisoara, Romania

## Abstract

**Introduction:**

SARS-Cov-2 pandemic resulted in a great amount of mental health suffering both in patients, families and medical staff.

**Objectives:**

To assess the personal impact of SARS-Cov-2 management in hospital health care staff.

**Methods:**

The study included 300 participants, comprising medical staff, 150 of which directly treated patients with SARS-Cov-2 infection, whilst the rest did not. Participants were asked to fill in online self-assessment scales: PSS-10 (Perceived Stress Scale), BAI (Beck Anxiety Inventory), BDI (Beck Depression Inventory), and SSOSH (Self Stigma of Seeking Psychology Help Scale). The data were collected at the end of 2021 during the COVID-19 pandemic with the Omicron variant.

**Results:**

238 (79.3%) women and 62 (20.7%) men responded to the online assessment. Participants working in COVID wards had higher scores for anxiety and depression (χ²=12.21, p=0.007). The intensity of depression and anxiety (BDI / BAI) depends on the professional degree (higher in senior specialists and specialists than in nurses and residents), working in shifts (χ²=8.77, p=0.01) and recent contact with patients infected with SARS-Cov-2 (χ²=76.10, p<0.0001). Regarding PSS-10 total scores, participants that had contact with 1-10 SARS-Cov-2 patients during the past month had significantly higher scores than those who did not. SSOSH showed that participants who had more than 5 on-calls per month had significantly higher scores than the participants who had one on-call per month (p=0.008). Logistical regression showed that participants who had higher BDI scores had a probability of 136.67 times higher (95% CI [16.42; 1137.53]) to present high stress levels.

**Conclusions:**

Respondents who had higher scores in the BDI (and not other assessment scales) had by far the highest probability of developing high stress levels.

**Disclosure of Interest:**

None Declared

